# Effectiveness of Convalescent Plasma Therapy in COVID-19 Patients with Hematological Malignancies: A Systematic Review

**DOI:** 10.3390/hematolrep14040052

**Published:** 2022-12-12

**Authors:** Sapha Shibeeb, Ilham Ajaj, Hadeel Al-Jighefee, Atiyeh M. Abdallah

**Affiliations:** 1 La Trobe College Australia, La Trobe University, Melbourne, VIC 3086, Australia; 2 Department of Biomedical Science, College of Health Sciences, QU Health, Qatar University, Doha 2713, Qatar

**Keywords:** convalescent plasma, COVID-19, SARS-CoV-2, hematological malignancy

## Abstract

**Background:** Immunocompromised patients, including those with hematological malignancies, are at a high risk of developing severe coronavirus disease 2019 (COVID-19) complications. Currently, there is a limited number of systematic reviews into the efficacy of convalescent plasma therapy (CPT) use in the treatment of COVID-19 patients with hematological malignancies. Therefore, the aim of this review was to systematically appraise the current evidence for the clinical benefits of this therapy in COVID-19 patients with hematological malignancies. **Methods:** A comprehensive search was conducted up to April 2022, using four databases: PubMed, Web of Science, Science Direct, and Scopus. Two reviewers independently assessed the quality of the included studies. Data collection analysis was performed using Microsoft Excel 365 and GraphPad Prism software. **Results:** 18 studies met the inclusion criteria; these records included 258 COVID-19 patients who had hematological malignancies and were treated with CPT. The main findings from the reviewed data suggest that CPT may be associated with improved clinical outcomes, including (a) higher survival rate, (b) improved SARS-CoV-2 clearance and presence of detectable anti-SARS-CoV-2 antibodies post CP transfusion, and (c) improved hospital discharge time and recovery after 1 month of CPT. Furthermore, treatment with convalescent plasma was not associated with the development of adverse events. **Conclusions:** CPT appears to be an effective supportive therapeutic option for hematological malignancy patients infected with COVID-19. To our knowledge, this is one of the first systematic reviews of the clinical benefits of CPT in COVID-19 patients with hematological malignancies.

## 1. Introduction

Since the first case report in December 2019, the severe acute respiratory syndrome coronavirus 2 (SARS-CoV-2), the causative agent of the coronavirus disease 2019 (COVID-19), has posed a significant challenge worldwide [1]. The clinical manifestation of COVID-19 ranges from having no signs or symptoms (asymptomatic) to severe complications that include thrombosis, septic shock, acute respiratory distress syndrome (ARDS), and cardiac failure [2]. Immunocompromised patients and cancer patients are among those who are at a high risk of a severe and prolonged disease course [3,4]. Hematological malignancies are heterogeneous blood cancers categorized according to the sites of origin—blood (leukemias), lymph nodes (lymphomas-Hodgkin and non-Hodgkin), or bone (myelomas) [5]. Patients with hematological malignancies represent a distinctive subset of those vulnerable to COVID-19 and were shown to be frequently associated with high mortality and COVID-19 complications [6]. Due to the underlying disease and cancer treatment, the immune system in these patients becomes impaired, thus making them immunodeficient and prone to infection and severe disease [2].

Convalescent plasma therapy (CPT) is a form of passive immunity where plasma enriched with specific and non-specific humoral innate immunity factors is collected from recovered patients, processed, and transfused into other patients [7]. During viral infection, the antibodies are key for virus opsonization and neutralization, in addition to the activation of complement and mediation of antibody-dependent cellular cytotoxicity. This type of treatment has been previously used to treat other infectious diseases such as Ebola, SARS, Middle East respiratory syncytial virus (MERS), and influenza [2]. Currently, COVID-19 has very limited treatment options, with isolation and supportive care being the major ones [8]. Some natural fruit and plant bioactive compounds have been shown to inhibit protease activity in SARS-CoV-2 [9]. In August 2020, the United States Food and Drug Administration (US FDA) issued an emergency use authorization (EUA) for COVID-19 convalescent plasma for the treatment of hospitalized patients with COVID-19 [10]. As a result, numerous trials have been conducted to assess the effectiveness of CPT in different COVID-19 patient cohorts including those with different disease severity and co-morbidities. The effectiveness of COVID-19 CPT in immunocompromised cancer patients, particularly those with hematological malignancies, has not been systematically reviewed yet. Therefore, we conducted a systematic review of the effectiveness of CPT to treat COVID-19 patients with hematological malignancies.

## 2. Methods

The study’s focus was on hematological malignancy patients (in remission and progression) with confirmed PCR COVID-19 infection. The intervention was CPT from previously infected COVID-19 patients. The control group had no CPT intervention, and studies with no control group were also included. The primary goal of this study was improvement in clinical outcomes, measured by survival outcomes and mortality, development of adverse events, and hospital discharge. The secondary goal was measured by viral clearance, defined as two consecutive negative RT-PCR test results 24 h apart and/or a decrease in RNAemia. SARS-CoV-2 RNAemia was measured using droplet-based digital RT-PCR (ddPCR) technology. We followed PRISMA guidelines in this search. The search was conducted up to June 2022 by two authors, using four major databases: PubMed, Web of Science, ScienceDirect, and Scopus. The following terms were used: ‘COVID-19’ or ‘SARS-CoV-2’ or Coronavirus’ AND ‘convalescent plasma’ or ‘convalescent plasma therapy’. All articles were extracted and assessed by two independent authors to ensure the quality of the research. All articles were exported to Endnote X9 and all duplicates were removed.

### 2.1. Inclusion and Exclusion Criteria

Articles were screened by title and abstracts. The following inclusion criteria were used for eligibility: (1) reported in English, (2) clinical trials including randomized and controlled clinical trials, and (3) prospective and retrospective comparative cohort studies, case-control studies, cross-sectional studies, case series, and case reports. Studies were excluded if they did not meet the inclusion criteria. In addition, all letters, editorials, systematic reviews, narrative reviews, abstracts, and non-full-text articles were excluded.

### 2.2. Quality Assessment and Risk of Bias Assessment

The risk of bias for all eligible observational studies was assessed according to the Strengthening the Reporting of Observational Studies in Epidemiology (STROBE) reporting guidelines by two authors, and if there was any discrepancy, it was resolved by consulting a senior author [11]. The risk was evaluated using a previously published question tool [12], which asked questions regarding (1) selection criteria of patients—namely, whether all the patients met the inclusion criteria; (2) adequate ascertainment of exposure and the outcome; (3) causality—namely, whether follow-up was long enough for outcomes to occur; and (4) reporting—namely, whether the case(s) were described with sufficient detail to allow other investigators to replicate the research or to allow practitioners to make inferences. All studies were scored for each question as: yes (2 stars), partial (1 star), and no (0 stars). An overall risk of bias was independently assigned to each eligible study by two authors. No studies rated below 5 were included in the systematic review (Table 1).

### 2.3. Data Extraction and Synthesis

The following clinical and laboratory variables were extracted: country, gender, age, type of hematological malignancy, cancer treatment, number of patients with CPT intervention, number of controls without CPT, and other treatments for COVID-19. Clinical outcomes including survival rate, adverse effects of CPT, and the titer of antibodies for donor and patient were recorded. Data analysis was qualitative: the collected data were interpreted based on the presence or absence of the clinical outcomes. The findings were collected using Microsoft Excel sheets.

### 2.4. Statistical Analysis

The clinical data that were extracted from the articles and assessed included survival, clinical outcome, viral clearance, seropositivity to SARS-CoV-2 antibodies, hospital discharge, CP dose, recovery period, development of adverse events to CPT, and companion treatments. The data analysis was only qualitative, and the collected data were interpreted based on the presence or absence of the clinical outcome. A meta-analysis was performed using GraphPad Prism Version 9.0 software to calculate the odds ratio and generate the forestplot. Dichotomous outcomes are presented as odds ration (OR) with 95% confidence intervals (CIs).

## 3. Results

### 3.1. Search Findings

**Figure 1:**
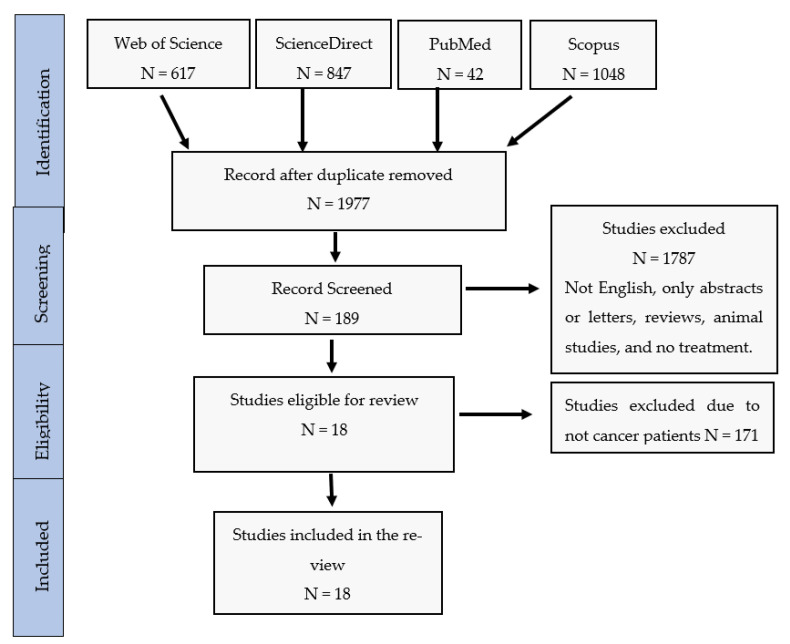
PRISMA Flow chart of study selection.

The outcome of the database search is described in a PRISMA flow chart (Figure 1). The search process yielded 2553 records, of which 578 articles were identified as duplicates and removed. Following title and abstract screening of the remaining 1975 articles, 1787 were excluded from the analysis due to one or more of the following reasons: (1) the article was published in a language other than English, (2) it was not an original research article, (3) it was an animal-based study, (4) the full-text was not available, or (5) the CPT was not used as a COVID-19 treatment. From the 188 full texts screened, 171 studies were excluded because they were not conducted on patients with hematological malignancies. Seventeen studies met the inclusion criteria and were used to perform this systematic review. We identified 1103 patients, of which 258 patients had one or more hematological malignancy, were diagnosed with COVID-19, and were treated with CPT, whereas 845 patients were included in the control group and were provided with standard care in two of the identified studies [15,20].

### 3.2. Study Characteristics and Patients’ Demographics

**Figure 2:**
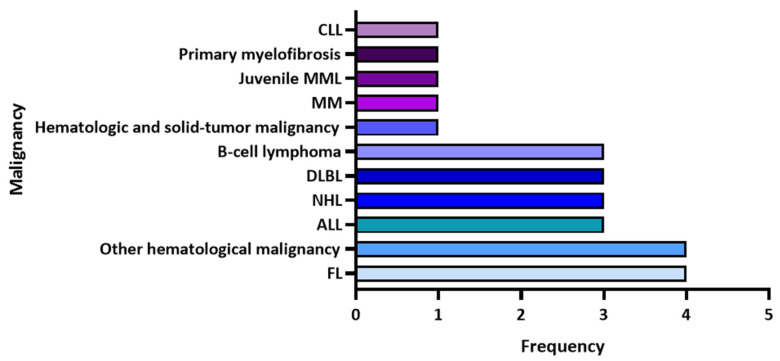
The frequency of hematological malignancies reported in the identified articles. CLL: chronic lymphocytic leukemia, MML: myelomonocytic leukemia, MM: multiple myeloma, HIV: human immunodeficiency virus, DLBL: diffused large B-cell lymphoma, NHL: non-Hodgkin’s lymphoma, ALL: acute lymphocytic leukemia, FL: follicular lymphoma.

Among the 17 selected articles, 13 were case reports or case series, two were retrospective cohort studies, and two were observational multicenter studies (Table 2). The reported hematological malignancies were mainly follicular lymphoma (*n* = 4), chronic lymphocytic leukemia ((*n* = 3), non-Hodgkin’s lymphoma (*n* = 3), diffused large B-cell lymphoma (*n* = 3), B-cell lymphoma (*n* = 3), and unspecified hematological malignancies (*n* = 4), (Figure 2). The range of patients’ ages was from 9 months to 72 years; the majority were older than 50 years while only three children were included (9 months and 4 and 5 years). A higher proportion of male patients were included in the studies compared to females (8:3). Ten patients had a history of receiving a stem cell transplant, of which seven were autologous transplants.

### 3.3. Dose, Time of Administration and Clinical Outcomes of CPT

The dose of CP varied from 200–300 mL per transfusion. CPT was administered at variable time-points after hospital admission, with some patients receiving CPT as early as day 2 post-diagnosis with COVID-19, while most of the remaining patients received CPT as a last treatment option after approximately one month of hospitalization (Table 3). In 14 studies, the total number of administered CPT doses ranged between 1–3 doses; however, two 53-year-old patients diagnosed with lymphoma were administered eight and 12 doses [2,18] due to their poor response to the initial treatment.

### 3.4. Assessment of Primary and Secondary Endpoints

**Figure 3:**
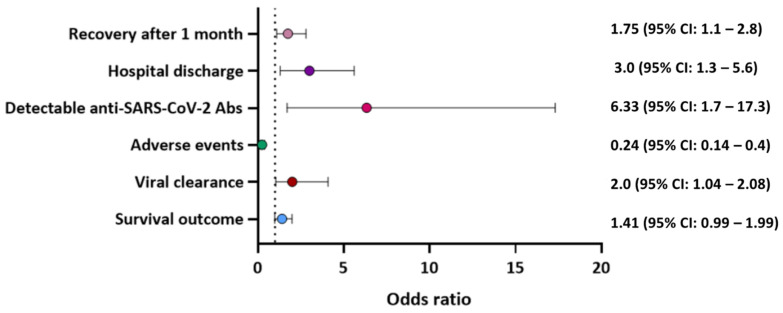
Odds ratio for the effectiveness of CPT on clinical outcome of patients with hematological malignancies and diagnosed with COVID-19.

The primary goal of this study was measured by survival outcomes and mortality, development of adverse events, and hospital discharge. Survival rate was improved following CPT treatment. Indeed, mortality was reported in 56 (21.7%) patients who received CPT compared to 213 (25.2%) control patients who received the standard care. Furthermore, the use of CPT was associated with improved overall survival (OR: 1.41, 95%CI: 0.99–1.99), hospital discharge (OR: 3.0 95%CI: 1.3–5.6), and recovery after 1 month of CPT (OR: 1.75 95%CI: 1.1–2.8). Moreover, the probability of adverse events in these patients due to CPT was significantly lower compared to the control group (OR: 0.24 95%CI: 0.14–0.40) (Figure 3). Adverse reactions to CPT were reported in only three patients who developed a febrile non-haemolytic transfusion reaction. Other treatments that were administered to patients along with CPT included oxygen therapy, steroids, Azithromycin, Hydroxychloroquine, and Remdesivir.

The secondary goal was assessed by viral clearance. It was found that the use of CPT aids in viral clearance (OR: 2.0, 95%CI: 1.04–2.08). Furthermore, detectable anti-SARS-CoV-2 antibody titer (IgG and/or IgM) was quantified by either a chemiluminescent microparticle immunoassay method (cut-off 1.40 index S/C) or using ELISA kit (cut off > 1.1). There were more detectable anti-SARS-CoV-2 antibodies after CPT than in the control detection (OR: 6.33 95%CI: 1.7–17.3).

## 4. Discussion

In this study, we report on the clinical benefits of using CPT to treat COVID-19 infected patients with hematological malignancy. Our analysis of the currently published studies that included appropriate control groups suggests that CPT may be associated with improved overall survival (OR: 1.41, 95%CI: 0.99–1.99), viral clearance, (OR: 2.0, 95%CI: 1.04–2.08), detection of anti-SARS-CoV-2 antibodies in the patient’s plasma post-CPT (OR: 6.33 95%CI: 1.7–17.3), and recovery after 1 month of CPT (OR: 1.75 95%CI: 1.1–2.8). Furthermore, the probability of adverse events due to CPT was low (OR: 0.24 95%CI: 0.14–0.40). Further studies with appropriate control groups are warranted to fully elucidate the effect of CPT on outcomes in COVID-19 patients with hematological malignancies.

The majority of the studies in the literature reported on the effect of CPT on COVID-19 outcomes in adult patients with hematological malignancies, and there are very limited published case reports in pediatric patients. Children with malignancies have always been at high risk of infections due to anti-cancer treatments resulting in an underlying immunocompromised state. Although several studies have reported less severe COVID-19 disease in children than adult cancer patients, there have been some reports of severe disease in children with cancer [7]. A few case reports have demonstrated improved outcomes following CPT in children with hematology malignancies [7,19,22].

A high mortality rate has been reported in hematological malignancy patients with severe COVID-19 [28]. The efficacy of CPT to reduce the mortality rate was reported in four of the 17 studies; however, only two studies compared the percentage of mortality to the control group. Thompson et al. reported a mortality rate of 13.1% in the CPT recipient group and 24.8% in the non-recipient group. Moreover, the mortality rate was significantly lower in intensive care unit patients on mechanical ventilation [15]. Furthermore, another study demonstrated a mortality rate of 13% among the CPT recipient group compared to a mortality rate of 41% in the non-recipient group [20].

A recent meta-analysis reported that using CPT could significantly reduce the risk of mortality in COVID-19 patients compared to those without CPT [29]. Jeyaraman et al. [25] and Tremblay et al. [8] reported a mortality rate of 45.5% and 35.7%, respectively. However, the efficacy of CPT to reduce mortality in these two studies is difficult to ascertain due to the lack of a control group. Abeldaño Zuñiga et al. [30] concluded that using CPT for hospitalized COVID-19 patients could result in clinical improvement for patients; however, it does not significantly lower mortality rates in comparison to standard care and placebo.

Most of the articles included in our analysis reported significant improvement in patient status after CPT, and patients become asymptomatic and were discharged. Four articles reported improved viral clearance after using CPT [19,22].

In all the reviewed articles, we found that CPT is safe, and no adverse reactions were reported in patients with hematological malignancies presenting with severe COVID-19 infection. However, one of the studies reported a febrile non-hemolytic transfusion reaction (FNHTR) in three patients post-CPT [8]. Supporting our findings, a recent meta-analysis concluded that CPT is a safe approach for COVID-19 patient therapy, with only 3.5% of recipients patients experiencing an adverse reaction [31]. Nevertheless, a systematic review stated that it is difficult to determine whether the adverse effect is due to the patient’s condition or CPT [32]. Therefore, more studies are required to investigate the safety of CPT in COVID-19 infected cancer patients.

A minimum of one ABO compatible CP dose was given to patients, with most receiving one to two doses. However, one patient with diffuse large B-cell lymphoma (DLBCL) who had a prolonged active COVID-19 infection for 129 days received eight doses of CP until remission [2]. There are no recommended or standardized doses of CP; however, most of the studies used one to two units, and the titer of the antibody might determine the optimal dose [33]. In our study, only four papers measured the titer of the donor antibodies before transfusion [8,19,22,26]. One of the included retrospective multicenter observational studies found that there were no significant differences in mortality in patients who received one versus two doses of CP or in patients who received early versus late transfusion of CP [25].

In all 18 studies, standard care including supportive care and antiviral therapy with hydroxychloroquine, azithromycin, and/or ritonavir, and Tocilizumab (for very critical patients) were given to patients before initiating CPT. In addition, most of the patients required oxygen therapy. Duan et al. reported that CPT combined with supportive care and antiviral therapy can improve the clinical outcome of COVID-19 patients [34]. Additionally, Agarwal et al. reported that using CPT alone might not be effective in reducing the severity, risk of mortality, and period of hospitalization of COVID-19 patients [35]. Therefore, it is recommended that CPT therapy be used as part of a combination of treatment to achieve the best result. To the best of our knowledge, this is one of the first systematic reviews to discuss the efficacy and safety of CPT in patients with hematological malignancies presenting with severe COVID-19 complications.

## 5. Limitations

Our meta-analysis has several limitations. First, the CPT treatment protocol had not been widely reviewed, and currently no clinical guidelines on the use of CPT, including the dose and donor antibody titer, are established for COVID-19 patients. Therefore, this has led to different CPT regimes. Furthermore, due to the lack of established clinical guidelines, CPT treatment outcomes are evaluated in multiple parameters.

Second, we were unable to perform the I2 statistic for heterogeneity because the number of studies included is small. Considerable heterogeneity may exist in this study due to the lack of an appropriate control group in most of the included studies and randomized control trials. Furthermore, the heterogeneity of CPT dose, time of transfusion, reported outcome such as viral clearance, donor antibody titers, and lack of control groups to compare the result made it difficult to perform a meta-analysis.

## 6. Conclusions

This review suggests that CPT may be used as a safe supportive therapy for patients with hematological malignancies and diagnosed with COVID-19 infection. The exact mechanism by which CPT may have mediated improved outcomes in the treated patients is likely multifactorial and could include reduction in viral load via enhanced clearance. Further studies and analysis are needed to fully understand the effectiveness of CPT in cancer patients with COVID-19.

## Figures and Tables

**Table 1:** Risk of Bias Assessment.

Author	Selection Criteria of Patients? *	Adequate Ascertainment Regarding the Exposure and the Outcome? *	Causality? *	Reporting? *	Total Score
Shankar et al. [7]	2	2	2	2	8
Szwebel et al. [13]	2	2	2	2	8
Wright et al. [14]	2	2	2	2	8
Tremblay et al. [8]	2	2	2	2	8
Thompson et al. [15]	2	2	2	2	8
Luetkens et al. [16]	2	2	2	2	8
Moore et al. [17]	2	2	2	2	8
Malsy et al. [18]	2	2	2	2	8
Rnjak et al. [2]	2	2	2	2	8
Balashov et al. [19]	2	2	2	2	8
Biernat et al. [20]	2	2	2	2	8
Çınar et al. [21]	2	2	2	2	8
Dell’Isola et al. [22]	2	2	2	2	8
Ferrari et al. [23]	2	2	2	2	8
Hueso et al. [24]	2	2	2	2	8
Jeyaraman et al. [25]	2	2	2	2	8
Karatas et al. [26]	2	2	2	2	8
Oliva et al. [27]	2	2	2	2	8

* Yes (2 stars), partial (1 star), and no (0 star).

**Table 2:** Summary of included studies on CPT for COVID-19 patients with hematological malignancies.

Author	Year	Country	Malignancy	Study Design	Sample Sizes CPT/Non-CPT	Age Median (Range)	Gender	Transplant
Shankar et al. [7]	2021	United States	ALL	Case report	1/0	4 years	F	
Szwebel et al. [13]	2021	France	HIV and cancer B-cell lymphoma	Case report	1/0	NA	M	ASCT
Wright et al. [14]	2021	United States	FL	Case report	1/0	54 years	M	
Tremblay et al. [8]	2020	United States	Hematologic and solid cancer	Case series	24/0	69 years (31–88)	41.7% F, 58.3% M	
Thompson et al. [15]	2021	Unites States	Hematologic cancer	Retrospective cohort study	143/823	65 years	NA	
Luetkens et al. [16]	2020	United States	MM	Case report	1/0	72 years	F	3 ASCT
Moore et al. [17]	2020	United States	NHL	Case report	1/0	63 years	F	
Malsy et al. [18]	2020	Germany Croatia	FL	Case report	1/0	53 years	F	
Rnjak et al. [2]	2021	Russia	DLBCL	Case report	1/0	53 years	M	ASCT
Balashov et al. [19]	2021	Russia	Juvenile myelomonocytic leukemia	Case report	1/0	9 months	F	HSCT
Biernat et al. [20]	2021	Poland	Hematological malignancy (Acute leukemia/MDS, CLL, Aggressive Lymphoma, MM)	Retrospective cohort study	23/22	NA	38% F, 62% M	
Çınar et al. [21]	2020	Turkey	MDS complicated by recently disseminated tuberculosis with associated kidney disease	Case report	1/0	55 years	M	
Dell’Isola et al. [22]	2021	Italy	B-cell ALL	Case report	1/0	6 years	F	
Ferrari et al. [23]	2021	Italy	FL	Case report	7/0	48 years	F	
			FL			60 years	M	
			Indolent NHL			60 years	M	SCT
			Primary myelofibrosis			43 years	M	
			DLBL			70 years	M	
			ALL			69 years	M	SCT
			CLL			60 years	M	
Hueso et al. [24]	2020	France	B-cell lymphopenia	Observational multicenter study	15/0	58 (35–77)	5 F, 12 M	
Jeyaraman et al. [25]	2021	India	Hematological malignancies	Retrospective observational multicenter study	33/0	62 years (18–80)	10 F, 23 M	1 ASCT
Karatas et al. [26]	2020	Turkey	Mixed cellularity classical Hodgkin lymphoma and peripheral T-cell lymphoma	Case report	1/0	61 years	M	ASCT
Oliva et al. [27]	2022	Italy	non-Hodgkin’s lymphoma	Retrospective observational single center study	6	59.5 years	6F	

CPT: convalescent plasma treated, CLL: chronic lymphocytic leukemia, MML: myelomonocytic leukemia, MM: multiple myeloma, HIV: human immunodeficiency virus, DLBL: diffused large B-cell lymphoma, NHL: non-Hodgkin’s lymphoma, ALL: acute lymphocytic leukemia, FL: follicular lymphoma. SCT: stem cell transplant, ASCT: autologous stem cell transplant, HSCT: hematopoietic stem cell transplant, MDS: Myelodysplastic syndrome, M: male, F: female.

**Table 3:** Summary of clinical outcomes from selected articles.

Authors	Sample Sizes CPT/Non-CPT	Day of CPT Administration	No. of CP Units (Dosage)	Outcome Endpoint	Outcome	Mortality	Adverse Events to CPT	Drugs
Shankar et al. [7]	1/0	Day 8 and 9 post illness onset	2 (15 mL/kg)	14 Days	Asymptomatic after 14 days	0%	None	Oxygen therapy, Steroids (hydrocortisone and dexamethasone)
Szwebel et al. [13]	1/0	Day 65 and 66 post symptoms onset	2 units daily	70 Days	Asymptomatic after 70 days	0%	None	Oxygen therapy, dexamethasone, oral prednisone, lopinavir/ritonavirm tocilizumab
Wright et al. [14]	1/0	NA	1 (200 mL)	1 month	Asymptomatic after 1 month and improvement of bilateral pulmonary infiltrates	0%	None	Azithromycin and HCQ, oxygen therapy and supportive care
Tremblay et al. [8]	24/0	Median time: 3 days between doses	2 (250 mL)	NA	13 patients were discharged home, 1 patient still hospitalized, and 10 patients died	41.7%	3 patients had FNHTR	Oxygen therapy and HCQ or azithromycin or remdesiviror tocilizumab or combination
Thompson et al. [15]	143/823	NA	-		Significantly improved 30-day mortality	13.3% vs. 24.8%	None	Corticosteroids, remdesivir, tocilizumab, and HCQ
Luetkens et al. [16]	1/0	NA	1 (200 mL)	6 days	Asymptomatic at approximately 6 days from onset	0%	None	Oxygen therapy
Moore et al. [17]	1/0	Day 88 post illness onset	1 (200 mL)	97 days	Asymptomatic at approximately 97 days from onset	0%	None	Metoprolol for heart rate regulation and apixaban for anticoagulation
Malsy et al. [18]	1/0	Day 85 post illness onset	Two-course of 6 units (2 units/day administered every other day	140 days	Asymptomatic at approximately 140 days from onset	0%	None	Remdesivir
Rnjak et al. [2]	1/0	Day 48, 49, 54, 55, 56, 57, 105 and 109 post illness onset	8 (~200 mL)	129 days	Afebrile with regression of pneumonia at 129 days from onset	0%	None	Oxygen therapy, remdesivir and steroids
Balashov et al. [19]	1/0	Day 146 post HSCT	3 (10 mL/kg)	4 months	Complete viral clearance, full resolution of the lung lesions on CT	0%	None	Tocilizumab and methylprednisolone
Biernat et al. [20]	23/22	Day 2–3 after diagnosis	1–2 (200–250 mL)	Day 14	Milder infection, less severe and faster resolution of symptoms, viral clearance	13.0% vs. 41.0%	None	Oxygen therapy, mechanical ventilation, HCQ, Dexamethasone, Remdesivir, Tocilizumab, Lopinavir/Ritonavir
Çınar et al. [21]	1/0	Day 5 post symptoms onset	2 (200 mL)	Day 7	Improved dyspnea and fever resolution, viral clearance	0%	None	Tocilizumab and favipiravir
Dell’Isola et al. [22]	1/0	Day 10 post admission	3 (10 mL/kg)	Day 18	Viral clearance	0%	None	Remdesivir and prednisone
Ferrari et al. [23]	7/0	NA	3 (210 mL)	Day 2	COVID-19 symptoms resolved, viral clearance, radiological improvement	0%	None	Corticosteroid, HCQ, low-molecular-weight heparin, and antibiotics
		NA		Day 2	COVID-19 symptoms resolved, viral clearance, radiological improvement			Corticosteroid, HCQ, low-molecular-weight heparin, and antibiotics
		NA		Day 3	COVID-19 symptoms resolved, radiological improvement			Corticosteroid, HCQ, low-molecular-weight heparin and antibiotics
		NA		Day 7	COVID-19 symptoms resolved, viral clearance			Corticosteroid, HCQ, low-molecular-weight heparin, and antibiotics
		NA		N/A	COVID-19 symptoms resolved, viral clearance, radiological improvement			Corticosteroid, HCQ, low-molecular-weight heparin, and antibiotics
		NA		Day 7	COVID-19 symptoms resolved, viral clearance, radiological improvement			Corticosteroid, HCQ, low-molecular-weight heparin, and antibiotics
		NA		Day 7	COVID-19 symptoms resolved, viral clearance, radiological improvement			Corticosteroid, HCQ, low-molecular-weight heparin, and antibiotics
Hueso et al. [24]	15/0	Day 0 + 1 (2 units each)	4 (200–220 mL)	Day 2	Fever resolved, and COVID-19 symptoms resolved after 2 weeks, decrease in RNAemia within 7–14 days	5.9%	None	Remdesivir and tocilizumab
Jeyaraman et al. [25]	33/0	4 days apart (range: 2–25 days)	1–2 (200 mL)	Day 3	Fever resolved	45.5%	None	HCQ, remdesivir, favipiravir, other broad-spectrum antibiotics, steroids (methylprednisolone or dexamethasone), tocilizumab and oxygen support
Karatas et al. [26]	1/0	Day 40 post admission	1	Day 34	Persistent SARS-CoV-2 viral shedding for 74 days	0%	None	HCQ and azithromycin
Oliva et al. [27]	6/0	51 post infection	3 (300 mL)	3–9 days	5 survived and 1 death	20%	1 Transient sinustachycardia	anti-CD20 drugs with different anti-viral medications for each patient

CPT: convalescent plasma treated, HCQ: Hydroxychloroquine.

## Data Availability

Not applicable.
